# Diamond lateral FinFET with triode-like behavior

**DOI:** 10.1038/s41598-020-59049-5

**Published:** 2020-02-10

**Authors:** Biqin Huang, Xiwei Bai, Stephen K. Lam, Samuel J. Kim

**Affiliations:** HRL Laboratories LLC, 3011 Malibu Canyon Road, Malibu, CA 90265 USA

**Keywords:** Engineering, Electrical and electronic engineering, Physics, Electronics, photonics and device physics

## Abstract

In this letter we report a diamond lateral FinFET fabricated using an ohmic regrowth technique. The use of ohmic regrowth separates the source/drain and gate fabrication, providing a viable means to improve ohmic contact resistance while protecting the top surface of the diamond channel from dry etch damage. Enabled by high channel quality, the diamond transistor behavior was shown to transit from a pentode-like to a triode-like characteristic when channel length decreased. For the first time, space charge limited transport in diamond FinFETs with a short channel length was demonstrated. We have analyzed the space charge limited transport from room temperature to 150 °C. This space charge limited transport, in combination with improved ohmic contacts, will enable diamond FinFETs for various high-power applications.

## Introduction

Diamond has attracted significant interest as an ideal material for RF and power electronics. Most studies have been focused on hydrogen-terminated FETs (HFETs) due to the ease of creating a p type conductive channel in diamond by hydrogen surface termination^1^. Although significant progress has been made in HFET, there are concerns regarding the long-term stability of two-dimensional hole gas and the low mobility^2^ at high carrier concentration. To overcome these limitations, we recently demonstrated a new device, diamond FinFET^3^, by leveraging the latest device concept in the silicon CMOS industry. Diamond fin structure enables the device to operate in accumulation or depletion modes without hydrogen termination. The previous work was built with a p+/p− diamond thin-film on a diamond substrate. To eliminate shorted source/drain, the top p+ diamond layer has to be selectively etched away. The plasma- based etch process inevitably damages the top surface of the fin channel, leading to potentially high surface defects and low channel mobility. To avoid these issues, we adopted a widely used ohmic regrowth technology^4^ for diamond FinFET. The ohmic regrowth approach eliminates the fin channel damage and enables the short channel devices for potential high-speed RF applications with improved ohmic contact resistance.

In a typical MOSFET, the channel current saturates with the increase of drain voltage due to the channel pinch-off, showing a pentode-like behavior. Continuously increasing drain voltage will move the pinch-off point toward the source. Once this pinch-off point reaches the source, the device current starts to increase, eventually leading to a regime in which space charge limited transport dominates. In this regime, the device behaves like a vacuum triode, where current is proportional to the square power of voltage bias^5^. This triode-like characteristic had been observed in various devices and materials such as Si^6^, SiC^7^, and recently GaN^8^. We demonstrated diamond FinFET devices with different transistor behaviors that benefited from the improved fin channel and ohmic contact. The space charge limited transport in diamond thin film had been studied^9^, but it was not demonstrated in a transistor until now. The triode-like behavior in diamond opens a new area for diamond electronics. Within this regime, the diamond device could provide high power capability without requiring a fully ionized channel. For certain applications, the channel could be intrinsic as long as the source/drain contact resistance is sufficiently low. That can be achieved by optimizing diamond regrowth.

## Results

**Figure 1:**
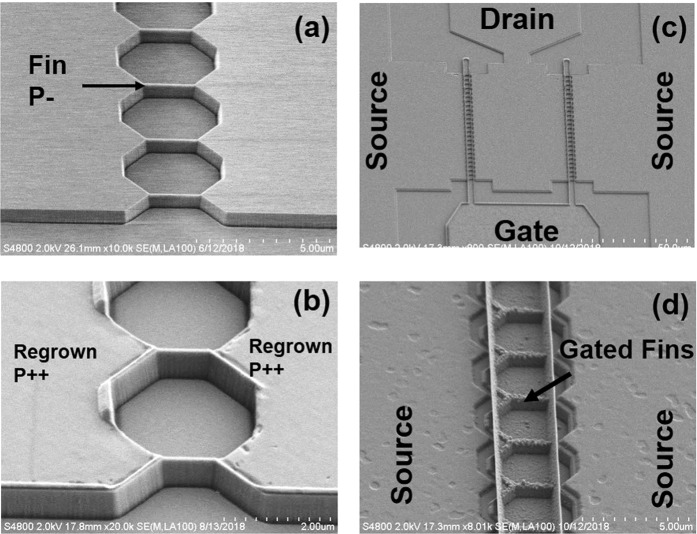
(**a**) The SEM image of diamond Fin channels fabricated in p- diamond layer. (**b**) Device after diamond regrowth, showing well protected fin channels. (**c**) Device after gate metal was fabricated, showing 25 fin channels with source (S), drain (D), and gate (G) labeled. (**d**) The zoom in image of the gated channel region. The fence like feature in the gate metal is due to the sputtering process.

The device was fabricated on a single crystalline diamond sample with 500 nm lightly boron-doped layer (P-) on top. The boron doping concentration in P- layer is nominally around 5 × 10^16^cm^−3^ and the substrate is nominally undoped. 100nm-wide 660 nm-tall fin structures were first formed through dry etching in O_2_/Ar(40sccm/10sccm) plasma as shown in Fig. 1(a). Following that a silicon oxide layer was deposited and patterns were formed by dry etching to expose the source and drain regions for diamond regrowth. Figure 1(b) shows the diamond surface after regrowth and the oxide layer removal. The process was optimized to minimize the height difference (~20 nm) between the ohmic region and the channel in order to reduce surface topography. SEM inspections indicate the relatively rougher surface and some defects in the regrowth region are likely caused by the etched defects in regrowth surface preparation. A completed device is shown in Fig. 1(c), featuring a 2 × 25 finger FinFET with source (S), drain (D), and gate (G) labeled. Figure 1(d) shows the gated channels with SiO_2_ and Al as gate dielectric and gate metal, respectively. Overall, the channels in this device are well preserved because of the regrowth process. The top of the fin channel is the original epitaxially grown surface, never exposed to any etch/growth processes.

**Figure 2:**
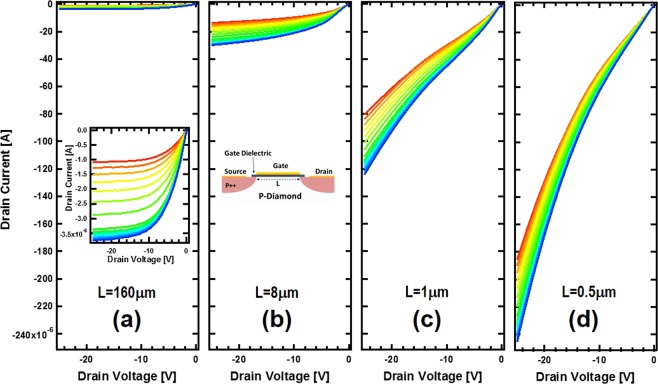
The IV transfer characteristics at room temperature for planar MOSFET with channel length varying from 160 μm to 0.5 μm. The gate voltage was swept from 5 V to −10 V during the test. (**a**) Inset is the replot of the same data for clarity. (**b**) Inset is the device structure, showing the source, drain and gate.

**Figure 3:**
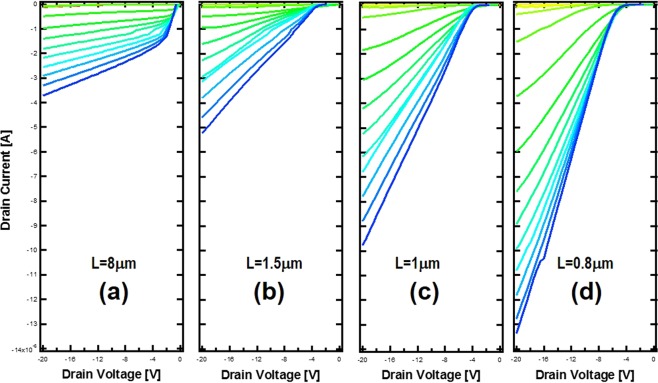
IV transfer characteristic at 150 °C for FinFET devices with channel varying from 8 μm to 0.8 μm, showing a transition from pentode like behavior to triode like behavior.

Planar MOSFET devices were fabricated on the same chip as FinFET devices for the hole transport study. The device structure is shown in Fig. 2(b) inset. Room temperature transfer characteristics for devices with channel lengths varying from 0.5 μm to 160 μm were measured as shown in Fig. 2. Figure 2(a) inset is a replot of the same IV curve for a 160-μm device at a different scale for clarity. All devices were tested with the gate bias swept from 5 V to −10 V relative to the grounded source. For these planar devices, 500 nm channel thickness makes it difficult to pinch off the device with small gate bias used in this test, as shown in all of these devices. For a longer channel device, the gate has better control over the channel so that the channel modulation is obvious as the Fig. 2(a) inset plot indicates, while for short channel devices the channel modulation becomes weaker because of the influence of drain voltage bias. Despite no pinched-off behavior, these planar devices with well-preserved top channel surface and much less impact from channel sidewalls provide an effective means to study the hole transport through bulk-like diamond. It can be seen there is a clear behavior transition from left to right in Fig. 2. For longer channels, the devices behave like a pentode, a typical MOSFET behavior. The drain current saturates at larger drain bias due to the formation of a depletion region next to the drain. For a short channel device, at the same drain bias, the edge of the depletion region originated from the drain is closer to the source region. At a certain point, the drain induced barrier lowering starts to cause non-saturated drain current as shown in Fig. 2(b). Once the depletion region reaches the source region, the channel current starts to increase significantly, as shown in Fig. 2(c,d). Ideally, the channel current will be an exponential function of the barrier between the source (P++) and channel (P−) like a pn junction if the channel can support the significantly increased current. However, due to much lower dopant concentration in the channel, the injected holes quickly form an internal electric field that limits the current injection from the source, leading to space charge limited transport as shown in Fig. 2(c,d) for large drain voltage. As discussed in our previous work^3^, to regain full control over the channel in diamond transistors with short channels, a fin structure is needed unless the p- layer thickness is below 100 nm or much lower doping is used. Current diamond growth technology is still not mature enough to achieve such thin film with reasonable quality. Figure 3 is the measured IV at 150 °C for FinFET devices fabricated on the same chips as the planar devices with channel varying from 0.8 μm to 8 μm. The same measuring condition as with the planar devices was used. As expected, the channel can be easily pinched off even for sub-micron devices. For small drain bias, short channel devices, as shown in Fig. 3(c,d), cannot be turned on even with large negative gate bias, different from the long channel devices in Fig. 2. This is likely due to the gate misalignment relative to the source, leaving a portion of the channel ungated. This ungated channel between source and gate will remain off until the drain-bias-induced depletion region reaches the source. In an ideal device, there will be an overlap between the gate and source, eliminating this ungated region. However, the pentode-like to triode-like transition is clearly observed in FinFET devices, as shown. This is consistent with observations in planar devices.

## Discussion

**Figure 4:**
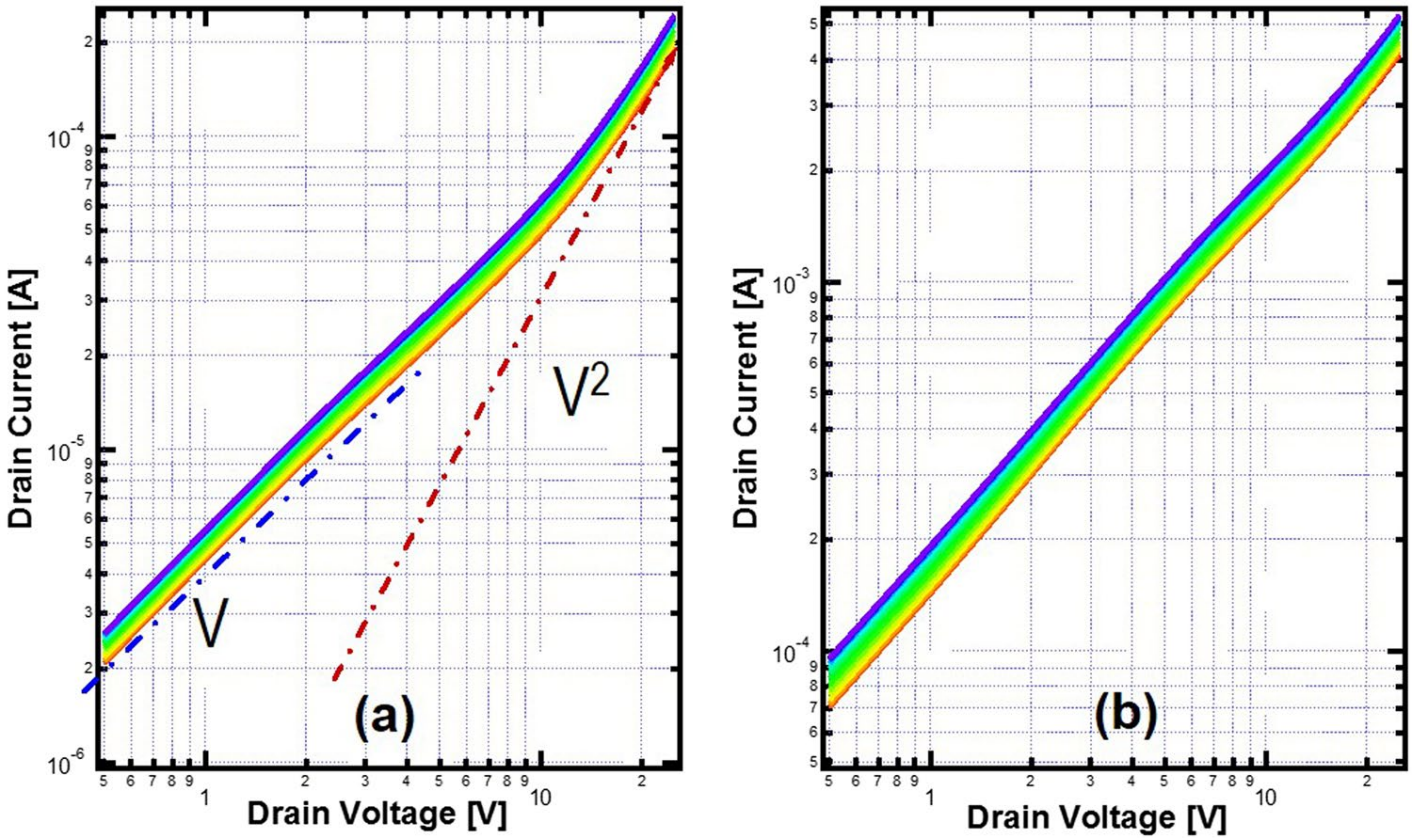
(**a**) The replot of IV data in log-log scale for the planar MOSFET with 0.5 um channel length in Fig. 2 (**d**). Lines indicating V or V^2^ relationships are guides for the eye. At low voltage, a linear ohmic behavior is observed while a square law behavior is observed in the high voltage bias regime. (**b**) The IV data of the same device tested at 150 °C shows a linear behavior only.

In Fig. 2, nonlinear IV characteristics were observed in planar MOSFETs, pointing to space charge limited transport in diamond. For current flowing in a doped semiconductor, assuming perfect ohmic contact, at small drain voltage the drain current follows ohmic law. Continuously increased drain voltage increases the carrier injection from the source. Once the injected carrier density is above the thermal carrier density provided by activated dopants, the current starts to deviate from the linear ohmic behavior. Eventually the current voltage characteristic can be described by the Mott-Gurney Law^10,11^: $I=\frac{9\varepsilon \mu A{V}^{2}}{8{L}^{3}}$, where I is the current, ε is the permittivity, µ is the mobility, V is voltage bias, L is the device length and A is the cross-section area for current. This is similar to electron transport in vacuum described by Child’s Law^12^. In Fig. 4(a), the IV data of Fig. 2(d) for the planar MOSFET with 0.5-um-long channel is replotted in log-log scale. In a low drain voltage regime, the drain current increases linearly as the drain bias, like a resistor. With the increase of drain bias, carriers injected from the source increase continuously. Eventually the injected hole density is significantly higher than the hole density provided by activated boron dopants in diamond, leading to space charge limited transport, as described by the theory developed by Mott and Gurney. Due to the partial ionization of boron dopant in diamond, the ionized boron in the channel is typically less than 1% of the boron concentration (nominally 5 × 10^16^/cm^3^). Lowly activated dopant concentration makes it easier to observe space charge limited transport since there are much less thermal carriers to overcome, as shown in Fig. 4(a). If the device temperature increases, more boron dopants will be activated, leading to a much more conductive channel. At the same time, the ohmic contact resistance is also reduced due to non-degenerated doping used in current ohmic regrowth. The same device was tested at 150 °C and the result is shown in Fig. 4(b). As expected, the current increases significantly from room temperature. However, the current-voltage relation stays linear within the tested voltage range. The cross-over voltage Vcr where current-voltage relation transits from Ohmic’s law to Mott-Gurney’s law can be found by using the method of region approximation^11^: ${V}_{cr}=\frac{4q{n}_{0}{L}^{2}}{3\varepsilon }$, where q is the single electron charge, n_0_ is thermal carrier density. It can be seen that the cross-over voltage is proportional to thermal carrier density which is the activated boron dopant concentration in diamond devices. Because of temperature dependent ionization, the transition voltage will be pushed to higher voltage bias at higher temperature. From room temperature to 150 °C, the activated boron dopant concentration increases approximately by more than 10×, consequently moving the transition voltage out of the test range. Hence, only ohmic behavior was observed.

In summary, diamond FinFET with ohmic regrowth process was demonstrated. The device behavior transition from pentode-like to triode-like was demonstrated by varying the channel length. The space charge limited transport in diamond transistor was observed for short channel devices. This space charge limited transport will be very useful for studying the charge transport in diamond, especially for studying the defects in diamond material. Beyond this, the diamond electronic device operating at a space charge limited transport regime could lead to new research areas where practical and powerful diamond electronic devices will be built for future power and RF electronics, similar to what has been explored in static induction transistors^13,14^.

## Methods

A (100) 10 × 10-mm^2^ undoped diamond substrate with an epitaxially grown p- layer was used. The p+ layer was regrown with microwave plasma CVD using a patterned SiO_2_ mask. Ti/Pt/Au was evaporated to form a good ohmic contact after 525 °C annealing in argon gas after regrowth and mask removal. 45 nm SiO_2_ gate dielectric was deposited by atomic layer deposition at 200 °C. To conformably wrap the gate around the sidewalls of the fins, Al metal was sputtered with a photoresist in place, then the metal was lifted off. Finally the ohmic contact pads were open with wet etching.
